# Physical Activity Decreases Somatic Symptom Distress in the Affect and Symptom Paradigm

**DOI:** 10.1097/PSY.0000000000001425

**Published:** 2025-08-07

**Authors:** Tara M. Petzke, Ferenc Köteles, Omer Van den Bergh, Michael Witthöft

**Affiliations:** Department of Clinical Psychology, Psychotherapy, or Experimental Psychopathology, Johannes-Gutenberg University Mainz (Petzke, Witthöft), Mainz, Germany; VIFASOM, Université Paris Cité (Petzke); Institut Psychiatrie et Neuroscience de Paris, INSERM U1266 (Petzke), Paris, France; Department of General Psychology and Methodology, Károli Gáspár University of the Reformed Church in Hungary (Köteles), Budapest, Hungary; Health Psychology, University of Leuven (Van den Bergh), Leuven, Belgium

**Keywords:** affect, persistent somatic symptoms, predictive processing, physical activity, symptom perception, **(Et)CO_2_** = (End-tidal) carbon dioxide, **(rm)AN(C)OVA** = (repeated measures) analysis of (co-)variance, **ASP** = Affect and Symptom Paradigm, **BPM** = beats per minute (aka heart rate), **CSD** = Checklist of Symptoms in Daily Life, **ETUDE** = Encompassing Training in fUnctional Disorders across Europe, **HiTOP** = Hierarchical Taxonomy of Psychopthology, **IBAc** = interoceptive breath counting accuracy, **JARS** = Journal Article Reporting Standards, **PHQ** = Patient Health Questionnaire, **RPM** = respirations per minute (aka respiratory rate), **SAM** = Self-Assessment Manikin, **TAc** = tone counting accuracy

## Abstract

**Objective:**

According to recent empirical taxonomies (eg, the Hierarchical Taxonomy of Psychopathology), somatic symptom distress represents a transdiagnostically relevant dimension of psychopathology. To better understand the mechanisms, somatic symptoms can be experimentally provoked by inducing negative affect (in the Affect and Symptom Paradigm, ASP, formerly known as the Affective Picture Paradigm). Potential moderators of this relation include cardiorespiratory activation and body-focused attention.

**Methods:**

In this preregistered, cross-sectional study (https://osf.io/sc57z/), we tested whether cardiorespiratory activation and body-focused attention modulate somatic symptoms in the ASP. Participants ( N=144 ) completed 3 ASPs, interlaced with cardiorespiratory activation (cycling) and rest. Participants were randomized to a body-attention or distraction condition. We measured heart rate, respiratory rate, and end-tidal CO_2_ during these tasks. The data were analyzed using AN(C)OVAs, *t* tests, and multilevel models.

**Results:**

Exercise significantly increased, and rest significantly decreased immediatesomatic symptoms ( $F_{\left(1.86,\;262.68\right)}=53.80,\;p<.001,\;\eta_{\mathrm{part}}^{2}=0.28$ ). High levels of somatic symptoms were significantly related to a sustained greater decrease in somatic symptoms in the ASP after cycling (vs. rest, $F_{\left(1,136\right)}=8.061,\;p=.005,\;\eta_{\mathrm{part}}^{2}=0.056)$ . No significant effect of the attention manipulation was observed ( ${F_{\mathrm{att}}}_{\left(1,\;141\right)}=\;0.52,\;p=.47,\;\eta_{\mathrm{part}}^{2}=\;0.004$ ).

**Conclusions:**

Rest and exercise temporarily modulate somatic symptom reports, and people with higher baseline somatic symptoms show the strongest beneficial effect of physical activity on symptom reports in the ASP. The findings are compatible with recent models of predictive processing and active inference.

## INTRODUCTION

Feeling “out of sorts” and being miserable—often, bodily symptoms and negative affect—go hand in hand. Obviously, the experience of bodily symptoms can dampen a person’s affective state.^1,2^ However, being in a bad mood also induces bodily symptoms, as has been shown by research using the Affect and Symptom Paradigm.^3^

Current models of symptom perception suggest explanations for this. For example, the predictive processing model^4–8^ posits that symptom experience results from an active (Bayesian) inference process in which a *prior*—a (not necessarily explicit) hypothesis about the bodily sensations—and the actual bodily sensations are integrated into a *posterior model* of the sensations representing the eventual experience of the symptom. The reliability of the prior and the actual bodily sensations determines their relative contribution to the eventual symptom percept. Especially when physiological signals are weak and unspecific, priors may dominate the symptom construction process.^7^ Because the posterior model of previous symptom episodes generate priors for new episodes, priors and bodily signals (in terms of afferent sensory input) may grow further apart, which can lead to persistent somatic symptoms or even to mental health problems, such as somatic symptom disorder.^9,10^

The latter happens particularly when applying a “Better Safe than Sorry” processing heuristic.^11^ In the face of potentially threatening bodily sensations, high threat sensitive individuals (those high on negative affectivity) may reduce the level of detail when processing sensory-perceptual aspects of the bodily sensations and “jump to conclusions” confirming prior expectations about somatic symptoms. This explains why persons with high habitual symptom reports in daily life and patients with functional disorders show a reduced correspondence between experimentally induced changes in the body and self-reported symptoms.^12^

In this study, we intend to explore central mechanisms behind somatic symptom distress (based on the predictive processing model) by testing potential moderators of somatic symptom perception in the Affect and Symptom Paradigm (ASP). In this paradigm, participants view negative pictures and subsequently rate their somatic symptoms. Comparing with a control stimulus (usually neutral pictures), this experimental setup typically provokes increases in somatic symptom reports, especially in high habitual symptom reporters and in patients with functional somatic syndromes.^3,13–16^

Interestingly, using psychometric bifactor models of somatic symptom distress,^17^ we found that the elevated symptoms behind the ASP effect are mainly of cardiorespiratory nature.^16^ This allows for interesting research questions, as the cardiorespiratory system can be easily manipulated, for example, through exercise, breath-pacing interventions, including meditative/mindfulness tasks. Adding a physical activity task to the Affect and Symptom Paradigm could lead to 2 potential scenarios regarding the somatic input. On the one hand, physical activity creates unspecific physiological and sensory noise, as many different physiological processes are happening at once (ie, next to the musculoskeletal system, the endocrine, nervous, cardiovascular, and respiratory systems are involved). Possibly, people with high negative symptom priors would experience exercise as aversive, as some of the incoming signals might seem to confirm the prior of having serious symptoms of a disease, such as a heart attack. In addition, trait neuroticism is associated with functional disorders,^18^ and meta-analyses have confirmed a small but significant negative link between neuroticism and physical activity.^19–21^ On the other hand, people with less negative priors might have very precise and neutral, perhaps even positive, expectations toward how it feels when they exercise. This way, even preexisting symptoms could be “explained away” by having done exercise, thus alleviating perceived symptom burden. In a previous study, we showed that enhancing the strength of the prior through repeating the symptom assessment increased the level of symptoms elicited by the ASP.^22^ Therefore, further research is needed to investigate how manipulating cardiorespiratory processes would affect the associated symptom experiences in the ASP.

Attention directed to the body can also modulate the effects of priors. It seems that different types of attention exist, and that their characteristics determine whether this modulation is beneficial or not.^23,24^ On the one hand, some self-directed forms of attention can lead to negative outcomes, including catastrophizing, heightened health anxiety, and more symptom reports.^25–27^ Potentially, this negative type of attention activates or strengthens symptom-related priors—for example, by casting doubt on the body’s health when physiological signals and symptoms are vague.^22^ On the other hand, a more positive form of attention toward the body can be beneficial. Mindfulness-based interventions were meta-analytically linked to more accurate perceived awareness of the body.^28^ Training interoceptive attention allocation led to an improvement of health-associated parameters, such as the upregulation of heart-rate variability,^29^ lower symptom reports,^30^ or responsiveness to physiological hunger/satiation cues.^31^ In exercise contexts, there can be a competition of cues between internal information, such as perceived fatigue, and external information, such as distractor cues given by the experimenters.^32^ For example, participants exercising on a treadmill listening to distracting (ie, non–body-related) sounds reported less fatigue and physical symptoms than participants hearing their own breathing sounds.^32^ In another study, however, this finding could not be replicated.^33^ Thus, the role of attention in contributing to somatic symptom experiences is complex and needs further study.

The present study was designed to investigate the effects of attention to the body (vs. distraction) and physical activity (vs. rest) on symptom perception, using the ASP. We chose physical activity as a potential modifier of cardiorespiratory activity, as it is easy to manipulate, feasible, and both physiology (“true signal” in the predictive processing model) and symptom experience (“posterior”) can be measured. In addition, measuring the symptoms evoked by physical activity introduces an interesting new dimension to the ASP. We hypothesized that people in a body-attention condition would report fewer net symptoms than people in the distraction condition, as attention would strengthen the precision of the physiological signal. We also predicted that participants would report fewer net symptoms after rest phases than activity phases, because there is less arousal or somatic input during rest.

## METHODS

### Transparency and Openness

This study’s design and analysis plan are preregistered at https://doi.org/10.17605/OSF.IO/SC57Z. We report how we determined our sample size, all data exclusions, all manipulations, and all measures in the study, and we follow JARS.^34^ Data and code are available at https://osf.io/jtgv3/. All nonphysical materials are publicly available. Data were analyzed using only the tools and programs listed below.

We obtained ethical approval in accordance with the 7th revision of the Helsinki Declaration from the local ethics commission at the Psychological Institute of Johannes Gutenberg University Mainz (Protocol 2023-JGU-psychEK-035). The current study is part of the innovative training network ETUDE (Encompassing Training in fUnctional Disorders across Europe; https://etude-itn.eu/), ultimately aiming to improve the understanding of mechanisms, diagnosis, treatment, and stigmatization of Functional Disorders.^35^

### Participants

We used G*Power 3.1.9.7 to determine the ideal sample size.^36^ When aiming for *d*=0.5 at 80% power with 3 repeated measures in 4 groups and correlations up to *r*=0.5, *N*=124 persons are required. To buffer for technical errors, dropouts, and other sources of attrition, we decided on *N*=150 as a stopping rule.

We originally wanted to only rerecruit persons who participated in an earlier study.^16,37^ Because of the weight restrictions of the apparatus, only former participants who self-reported their weight at ≤95 kg were eligible.

However, we were unable to reach enough former participants; therefore, we recruited new participants via social media posts, posters around the city, newsletters, and press outlets. For these new participants, the inclusion criteria were being between 18 and 65 years old, weighing maximally 95 kg, and sufficiently understanding German.

The total recruitment and data collection period spanned from November 2023 to April 2024.

### Materials

#### Questionnaires

In this study, participants were asked to fill in the PHQ-15 questionnaire in advance online before their appointment with the experimenters. For this, we used the online platform SoSci Survey.^38^ The CSD and the SAM are integral parts of the ASP and were therefore implemented in Inquisit.^39^

**Patient Health Questionnaire** **—15 (PHQ-15)**. The patient health questionnaire measures symptom burden in the last 4 weeks.^40^ It consists of 15 items which are answered on a scale of 0 (not bothered at all) to 2 (bothered a lot). The PHQ-15 is considered a reliable and valid questionnaire, as it has been tested in many different settings.^41,42^ The German version is by Löwe et al.^43^ We measured an internal consistency of α=0.773​​.

**Checklist of Symptoms in Daily Life (CSD)**. The CSD was designed by Wientjes and Grossman^44^ to assess the severity of 25 selected symptoms. The items are responded to on a scale of 1 (not at all) to 5 (very strongly). We chose to only present 12 items of the CSD which had provoked the most net symptoms (after negative minus after neutral trials) in earlier studies,^16,22^ see also Supplemental Digital Content, Supplement A, http://links.lww.com/PSYMED/B117). The first CSD in the experiment had an internal consistency of α=0.755​​.

**Self-Assessment Manikin (SAM)**. The SAM is a low-verbal assessment instrument for valence and arousal designed by Bradley and Lang.^45^ Per item, it consists of 5 schematic drawings of a manikin displaying low to high valence/arousal. Participants can rate their current moods using a 9-point scale anchored just below the drawings.

#### Affect and Symptom Paradigm

**FIGURE 1:**
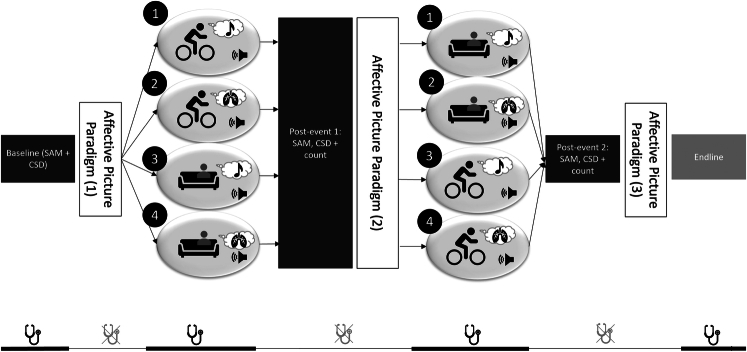
Procedure of the experiment with the 4 conditions*. Note*. Symptoms, valence, and arousal were assessed at baseline and each postevent (dark gray rectangles). No further questions were asked at the endline, but physiological measures were assessed for another minute. The timeline at the bottom of the figure indicates during which tasks physiological measurements were taken. When accounting for both the within-person and the between-person factors, there were 4 conditions: activity first–distraction; activity first–attention, rest first–distraction; and rest first–attention. Thus, attention versus distraction was a between-subject condition and activity versus rest was a within-subject condition. CSD=Checklist of Symptoms in daily life; SAM=Self-Assessment Manikin.

The Affect and Symptom Paradigm (ASP), also known as the Affective Picture Paradigm, was originally used by Bogaerts et al^3^ to demonstrate the influence of negative stimuli on symptom experience. Here, we used 24 negative and 24 neutral pictures from the International Affective Picture System (IAPS),^46^ Each trial consisted of 6 pictures of one valence level, which were presented for 7 seconds each (ie, the length of blocks was 42 s). After viewing a block of pictures, participants were asked about their own current valence and arousal using the SAM. Then, they were also asked about the intensity of current symptoms using the CSD. Negative and neutral trial blocks alternated, starting with negative trials, as we had determined that this was the best order.^16^ In summary, there were 4 negative and 4 neutral blocks per ASP task. Participants completed this task 3 times (Figure 1 and Supplemental Digital Content, Supplement B, http://links.lww.com/PSYMED/B117).

#### Attention/Distraction Tasks

Attention/distraction was a between-subjects condition. This task was modelled after the memory task by Van den Bergh et al.^47^ Participants were presented a series of high and low tones (piano note C_4_ and B_4_), which were randomly played at a ratio of 1:3. Between the notes was a random jitter, spanning 3000, 4000, or 5000 ms (ratio of 2:1:1). This way, there were exactly 16 tones presented per minute, which is similar to the average resting respiratory rate.^48^ Participants in the distraction condition were asked to count the high notes and disregard the low tones, while participants in the attention condition were instructed to ignore the tones and count their breaths. Thus, people in the distraction condition were doing a nondemanding working memory task with an external attention allocation, while people in the attention condition were doing a similar task with an internal attention allocation, to use Pennebaker and Lightner’s^32^ terms. After the task, participants from both conditions were asked how many tones/breaths they counted. All participants did this task twice—once while resting and once while cycling (physical activity).

#### Physical Activity Versus Rest

Activity and rest were within-person conditions, and the order was randomized (Figure 1). For the rest, participants were instructed to find a “relaxed and cozy seating position” and were offered to switch from sitting in the regular chair to an armchair. For the physical activity task, participants were instructed to get onto the bike ergometer and cycle at a self-chosen speed that was adequate for them. Participants were told that the task was not about maximal exertion, but that they should feel like they were doing some exercise.

Both resting and activity had a duration of 5 minutes, during which we recorded end-tidal CO_2_ (EtCO_2_), heart rate (BPM), and respiratory rate (RPM) 6 times—at the very beginning and after every minute. There were no additional resting times between physical activity (or rest) and the next questions, which were about current symptoms, valence, and arousal.

### Apparatus

For the physical activity task, we used a Wahoo Kickr Snap^49^ with a generic all-terrain bicycle with 24” wheels. Bike resistance was set to the seventh gear on the third chainring of a Shimano Freewheel 14-28T cassette.^50^ This corresponds to a speed of 13.8 km/h at 40 revolutions per minute, or 5.7 m per crank revolution. Participants were not allowed to change gears or adjust the resistance of the instrument.

We measured 3 types of physiological data using the PC-900B handheld capnograph and pulse oximeter.^51^ These measures, EtCO_2_, measured in mm Hg, heartbeats per minute (BPM), and breaths per minute (RPM), were added as a manipulation check. EtCO_2_ represents the partial pressure of CO_2_ in the total expiratory gas mix. It is one of the main drivers of respiratory regulation.^52^ An EtCO_2_ value of below 35 mm Hg is considered hypocapnia and is mostly caused by hyperventilation.

### Design and Procedure

We contacted former participants by sending an anonymized email through SoSci Survey. Former participants were asked to contribute to a follow-up study to the original study and were provided with a personalized link to an online survey. In contrast, new participants got the link through a QR code on a poster or by using a nonpersonalized link. After giving informed consent, participants first filled out demographic and health-related questions, followed by the above-mentioned questionnaires. At the end of the survey, the participants were redirected to a calendar tool to schedule a lab appointment with their anonymous code. Participants were sent a reminder of their participation up to 2 days before their appointment.

On the day of the lab appointment, participants were randomized into the attention or into the distraction condition. Within-person, the order of activity and resting was also randomized, resulting in a 2×2 within-between design: activity first–distraction; activity first–attention, rest first–distraction; and rest first–attention. So, attention versus distraction was a between-persons manipulation, while activity versus rest was manipulated within persons (Figure 1). When the participant arrived, we first assessed the baseline of 3 physiological parameters, that is, EtCO_2_, RPM, and BPM. These values were noted down twice: once immediately, and once after 1 minute of rest. After that, people were asked about their current symptoms (CSD), valence, and arousal (2 SAMs). Then, the participants completed the first run-through of the ASP, followed by the rest of the activity task, respectively. After answering the questions associated with the tasks (CSD and SAMs), the participants completed the second run-through of the ASP, followed by the remaining task (activity or rest), where we again recorded the physiological parameters 6 times. Participants also again filled in the CSD and SAMs. Following the third and last run-through of the ASP, we assessed the 3 physiological parameters again twice over the course of 1 minute. This procedure is also summarized in Figure 1.

After this, participants were debriefed and asked again for consent to use their data. Participants in the attention conditions were furthermore given the opportunity to redo the physical activity task, this time with distraction instructions, as we had hypothesized that this would be the more favorable condition. Last, participants were remunerated with 20€ or course participation credits.

### Statistical Analysis

We used SPSS Version 27^53^ and R Version 4.1.2^54^ for statistical analyses. Within the R environment, we used packages foreign, psych, ggplot2, lme4, and lmerTest.^55–60^ First, we removed duplicates and then reconstructed from which sources participants came and where they dropped out. After removing one person where the experimental protocol was violated, we calculated indices of interest, specifically net symptoms (see below) and interoceptive breath counting accuracy, as inspired by Schandry’s^61^ interoceptive heartbeat counting accuracy: $\mathrm{IBAc}=\frac{1}{k}\sum 1-\frac{\left|\mathrm{recorded}-\mathrm{counted}\right|}{\mathrm{recorded}}$ . Tone counting accuracy (TAc) for people counting tones in the distraction condition was calculated with the same formula.

As preregistered, we then conducted randomization checks using *t* tests and ANOVAs. Manipulation checks were conducted using the same methods—here, we tested whether negative and neutral pictures provoked different levels of symptoms, and whether the physiological measures fit our interventions. We expected EtCO_2_, BPM, and RPM to be highest after cycling. Our hypothesis testing consisted of repeated measures ANOVAs with before-intervention/after-rest/after-activity as the repeated measure, and order and distraction versus attention as between-factors. First, we did this with *net* symptoms (symptoms after negative minus after neutral trials) in the 3 ASP *blocks* as a main outcome; then, we calculated *event*-based rmANOVAs—*absolute* valence, arousal, and symptoms at baseline, immediate post-rest, and immediate postactivity (in Figure 1, these are depicted as black vertical rectangles). We used the PHQ-15 score as a covariate where appropriate, but in line with Wysocki et al’s arguments,^62^ we did not control for demographic variables.

Furthermore, we conducted some not-preregistered exploratory analyses, such as 2 multilevel models. We first conducted a linear regression to assess the feasibility of a hierarchical model. After centering net symptoms, we sequentially modelled an unconditional means model, the 2 unconditional growth models (random intercepts of time; random intercepts and random slopes of time), and then stated modelling conditional growth models. These contained the level-2 variables attention/distraction and order. All models were full maximum likelihood models. Models were sequentially tested against each other using a Likelihood ratio test.

## RESULTS

### Demographics

We were able to recruit n=234 persons who started filling out the online survey. Of these, *n*=33 did not finish the survey, *n*=15 were ineligible due to weight reasons, *n*=2 persons actively declined to participate after the survey, and *n*=39 did not come to a lab appointment. Due to an experimental protocol violation, one additional person had to be excluded from the analyses, which means our total sample size was *N*=144. All demographic data and other relevant characteristics can be seen in Table 1.

**TABLE 1:** Demographic Data and Participant Characteristics

	*M* (SD) or *N* (%)
Sex (male/female/other)	40/103/1
Age, y	28.69 (11.35)
Education	
No finished school form	1 (0.7%)
Low-level high school diploma	2 (1.4%)
Mid-level high school diploma	6 (4.2%)
High-level high school diploma	76 (52.8%)
Tertiary degree	59 (41.0%)
Occupation	
Student/ in vocational training	100 (69.4%)
Employed	29 (20.1%)
Civil servants	3 (2.1%)
Self-employed	3 (2.1%)
Jobseeking/unemployed	5 (3.5%)
Retired	4 (2.8%)
Body mass index	23.11 (3.18)
Somatic Symptom Burden (measured by PHQ-15)	5.97 (3.90)
People with moderate or severe symptom burden (PHQ-15≤10)	28 (19.4%)
Anxiety and depressiveness (measured by PHQ-4)	3.08 (2.49)
People with moderate or severe anxiety/depression (PHQ-4≤6)	21 (14.6%)
Alexithymia (measured by TAS-20)	42.47 (11.08)
People with alexithymia (TAS-20≤61)	11 (6.9%)
Self-reported psychological problems	30 (20.8%)
Depressive disorder	18 (12.5%)
Anxiety disorder	1 (0.7%)
Other	15 (10.4%)
Self-reported structural physical conditions	33 (22.9%)
Neoplasms	2 (1.4%)
Endocrine, nutritional, and metabolic diseases	7 (4.9%)
Diseases of the eye and adnexa	3 (2.1%)
Diseases of the circulatory system	4 (2.8%)
Diseases of the respiratory system	5 (3.5%)
Diseases of the digestive system	4 (2.8%)
Diseases of the skin and subcutaneous tissue	2 (1.4%)
Diseases of the musculoskeletal system & connective tissue	10 (6.9%)
Diseases of the genitourinary system	1 (0.7%)
Congenital mal-/deformations, chromosomal abnormalities	2 (1.4%)
Not elsewhere classified symptoms	2 (1.4%)
Allergies	2 (1.4%)
Self-reported functional conditions	33 (22.9%)
IBS	13 (9.0%)
Fibromyalgia	1 (0.7%)
Headaches	8 (5.6%)
Chronic fatigue	1 (0.7%)
Tinnitus	6 (4.2%)
Back pain (<3 mo)	11 (7.6%)
Premenstrual syndrome	6 (4.2%)
Other	3 (2.1%)

*Note*. IBS = irritable bowel syndrome; PHQ-15 = Patient Health Questionnaire 15; PHQ-4 = Patient Health Questionnaire 4, a very short screening tool for anxiety and depression by Kroenke et al^63^; TAS-20 = Toronto Alexithymia Scale, a questionnaire measuring alexithymia.^64^

### Manipulation Checks

In the ASPs, there was a stimulus material main effect ( $F_{\left(1.76,251.57\right)}=44.40,\;p<.001,\;{\eta^{2}}_{\mathrm{part}}=0.237$ ). Post hoc analyses revealed that negative pictures evoked significantly stronger somatic symptom reports than neutral pictures, but not more than at baseline. As we were surprised by the lack of difference between baseline and negative pictures, we looked at the symptom reports per trial. The trial-by-trial values suggested that absolute symptom reports in the CSD of the ASPs decreased over the course of the experiment and therefore obscured differences between the one baseline measurement and the mean of 12 gradually decreasing symptom reports after negative trials. Therefore, we conducted a multilevel model.

The analyses for physiological measures are described in detail in Supplemental Digital Content, Supplement C, http://links.lww.com/PSYMED/B117. The physiological measures confirmed a successful manipulation: EtCO_2_ and heart rate were highest while cycling (EtCO_2_: $M_{\mathrm{act}}=39.76,\mathrm{SE}=0.39,$ heart rate: $M_{\mathrm{act}}=88.45,\mathrm{SE}=1.55$ ). This suggests a successful, albeit light, physical activity manipulation.

Lastly, breaths per minute differed significantly between events ( $F_{\left(2.76,385.94\right)}=23.57,$ *p* $<.001,\;\eta_{\mathrm{part}}^{2}=0.14$ ). Again, people breathed the most during physical activity ( $M_{\mathrm{act}}=15.44,\;\mathrm{SE}=0.35,\;95\%\;\mathrm{CI}=\left[14.74,\;16.13\right]$ ), which was higher than all other events at the *p*=.001 level. There was a significant between-person effect of attention/distraction, as well as an event×attention/distraction interaction (between: $F_{\left(1,140\right)}=32.64,\;p<.001,\;\eta_{\mathrm{part}}^{2}=0.19$ ; interaction: $F_{\left(2.76,\;385.94\right)}=29.71,\;p<.001,\;\eta_{\mathrm{part}}^{2}=0.144$ ). People in the distraction condition breathed more frequently than people in the attention condition. There were no significant order effects, nor an event×order interaction.

People in the attention condition had an average breath counting accuracy of IBAc=0.75 (SD=0.72), that is, 75% of breaths were counted; the median was IBAc=0.90. In the distraction condition, people scored a mean of TAc=0.88 (SD=0.36), with a median of 0.98. This implies that people indeed paid attention to the stimuli they were instructed to pay attention to.

### Correlations

Baseline EtCO_2_ was inversely associated with PHQ-15 sum score ( $r=-0.23,\;95\%\;\mathrm{CI}=\left[-0.38;-0.07\right],\;p=.006$ ), while being independent of baseline CSD ( $r=-0.05,\;95\%\;\mathrm{CI}=\left[-0.21,\;0.11\right],\;p=.547$ ).

### Hypothesis Testing

Randomization checks are reported in Supplemental Digital Content, Supplement D, http://links.lww.com/PSYMED/B117. The analyses for valence and arousal are reported in Supplemental Digital Content, Supplement C, http://links.lww.com/PSYMED/B117. In summary, for event-based valence, the results were mostly insignificant. People reported the least amount of arousal after resting.

Regarding the net symptoms over the 3 ASPs, the between-subject effects of order and attention versus distraction were not significant ( ${F_{\mathrm{order}}}_{\left(1,141\right)}=2.67,\;p=.10,\;\eta_{\mathrm{part}}^{2}=0.019;\;F_{{}_{att}\left(1,141\right)}=0.52,\;p=.47,\;\eta_{\mathrm{part}}^{2}=0.004$ ). The time effect was significant ( $F_{\left(2,282\right)}=30.32,\;p<.001,\;\eta_{\mathrm{part}}^{2}=0.126$ )—the difference between negative and neutral pictures decreased over time. There were no interactions between net symptoms and order or attention/distraction ( ${F_{\mathrm{sympt}\times \mathrm{att}}}_{\left(2,282\right)}=0.07,\;p=.94,\;\eta_{\mathrm{part}}^{2}<0.001;\;F_{\mathrm{sympt}\times \mathrm{order}}\left(2,282\right)=0.99,\;p=0.37,\;\eta_{\mathrm{part}}^{2}=0.007$ ). Thus, there were no crossover effects, but also no influence of the attention condition on symptom ratings.

In the event-based rmANOVA with absolute CSD symptoms, the 3 timepoints differed significantly ( $F_{\left(1.86,\;259.72\right)}=53.59,\;p<.001,\;\eta_{\mathrm{part}}^{2}=0.28$ ). Participants reported most symptoms after activity ( $M_{\mathrm{act}}=16.99,\;\mathrm{SE}=0.31,\;95\%\;\mathrm{CI}=\left[16.38,\;17.59\right]$ ), followed by baseline ( $M_{\mathrm{base}}=15.34,\;\mathrm{SE}=0.28,\;95\%\;\mathrm{CI}=\left[14.78,\;15.90\right]$ ), and the least after rest ( $M_{\mathrm{rest}}=14.12,\;\mathrm{SE}=0.22,\;95\%\;\mathrm{CI}=\left[13.70,\;14.55\right]$ ). Post hoc comparisons revealed that these 3 events significantly differed pairwise at *p*<.001 each. The between-factors attention and order were both not significant ( $F_{\left(1,140\right)}=0.07$ and 0.37, *p*=.79 and .55, $\eta_{\mathrm{part}}^{2}=<0.001$ and 0.003, respectively). However, order interacted with event ( $F_{\left(1.86,\;259.72\right)}=9.65,\;p<.001,\;\eta_{\mathrm{part}}^{2}=0.064$ )—people in the activity-first order had more absolute somatic symptoms in the CSD after activity, and people in the rest-first condition had more symptoms after rest.

### Contribution of Habitual Symptom Reporting

Next to the above-mentioned repeated-measures ANOVAs, we explored the contribution of habitual symptom reporting as measured by the PHQ-15 on ASP effects. To fully focus on the effects of attention versus distraction and activity versus rest, we chose the 2 ASP run-throughs *after* the interventions as dependent repeated measures variables, order and attention condition as between-subjects factors, and PHQ-15 score, as well as effects of the first run-through as covariates.

**FIGURE 2:**
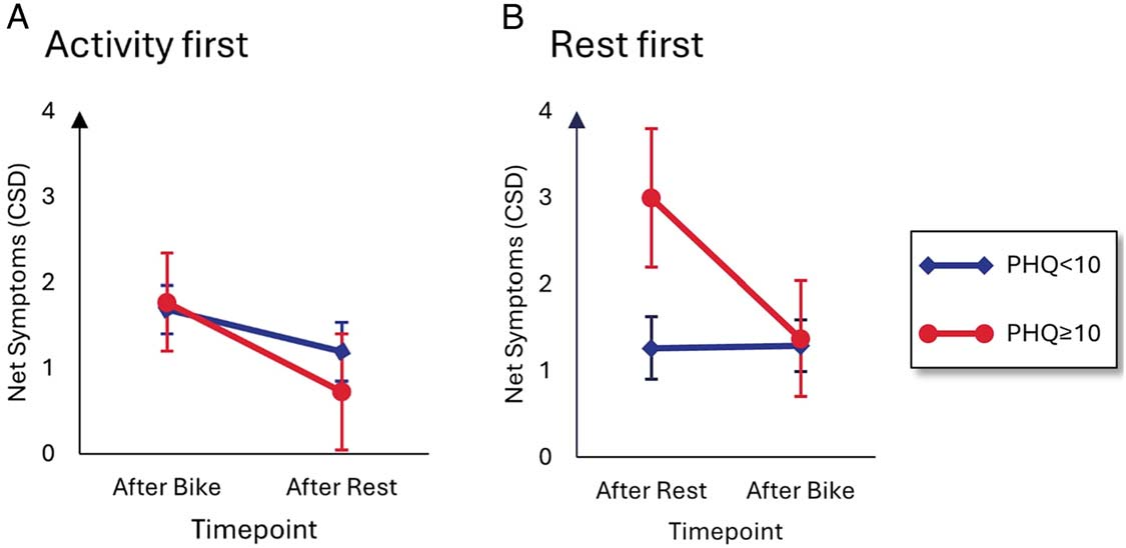
Symptom report by group and habitual symptom reporting. *Note*. Error bars represent 95% confidence intervals. In total, *n*=78 persons were in the activity-first condition (Panel A), while *n*=66 were in the rest-first condition (B). CSD= Checklist of Symptoms in Daily Life; PHQ-15 =Patient Health Questionnaire 15. Color image is available only in online version.

On a within-subjects level, the repeated measures effects were significant, $F_{\left(1,136\right)}=5.16,\;p=.025,\;\eta_{\mathrm{part}}^{2}=0.037$ . People showed higher ASP effects after cycling than after resting. None of the 2-way interactions showed significant associations (all *p*>.23, all $\eta_{\mathrm{part}}^{2}$ <0.01). The 3-way interaction between repeated measures, order, and PHQ-15 score was significant, $F_{\left(1,136\right)}=8.06,\;p=0.005,\;\eta_{\mathrm{part}}^{2}=0.056$ . Figure 2 shows that people with higher PHQ-15 scores (ie, above the clinically relevant cutoff of 10) have higher levels of net symptoms, which seem to reduce once they do the activity task. If they start with the cycling task, their net symptoms are at average levels and even drop slightly in the blocks after resting. The other 3-way and the 4-way interaction were not significant (both *p*>.34, all $\eta_{\mathrm{part}}^{2}<$ 0.008).

Between individuals, the first ASP run-through was a significant predictor of the later ASP effects, $F_{\left(1,136\right)}=270.81,$ *p* < .001, $\eta_{\mathrm{part}}^{2}=$ 0. 67 . People with higher levels of net symptoms in the first ASP run-through had higher net symptoms in the other ASP run-throughs. Higher PHQ-15 levels were also significantly associated with ASP effects, $F_{\left(1,136\right)}=4.56,$ *p* $=.035,\;\eta_{\mathrm{part}}^{2}=0.032$ . There was also an effect of the interaction of order and PHQ-15, $F_{\left(1,136\right)}=5.42,\;p=.02,\;\eta_{\mathrm{part}}^{2}=0.038$ . This mirrors the above-described within-person interaction between repeated measures, order, and PHQ-15 score.

### Time-Mapping the Net Symptom Reports

We conducted a multilevel analysis to check whether net symptoms changed over the course of the experiment. First, we ran a linear regression with just time. As this regression showed a significant influence of time on net symptoms ( b=−0.09,SE=0.02,p<.001 ), we continued to model the time effect using a hierarchical regression model.

The unconditional means model revealed that the intraclass correlation was 50.2%. Adding the random intercept of time, we discovered that 2.9% of the variations in net symptoms can be explained by the main effect of (linear) time ( b=−0.09,SE=0.01,p<.001 ). Next, we added random intercepts of time, which showed that 7.0% of net symptoms can be explained by the main and random effects of time ( b=−0.09,SE=0.02,p<.001 ).

Next, we built a conditional growth model with attention/distraction, order, and all interactions (3 two-way and 1 three-way interaction). However, this model was not significantly better than the second unconditional growth model, likely because none of the newly added predictors was significant (Supplemental Digital Content, Supplement E, http://links.lww.com/PSYMED/B117).

After that, we added PHQ-15 as an additional level 2 predictor of net symptoms. This model included time, PHQ-15, attention/distraction, order, as well as the 3 two-way and the three-way interactions of attention/distraction, time, and order. PHQ-15 total score predicted symptoms positively, meaning that people with higher habitual symptom reporting generally report more symptoms. By adding a random intercept for the PHQ-15 scores, an additional *R* ^2^=40.6% of the variance in intercepts can be explained. This model performed significantly better than the second unconditional growth model ( $\chi_{\mathrm{dif}}^{2}=52.85,\;\mathrm{df}=10,\;p<.001$ ).

### Additional Exploratory Analyses

In Supplemental Digital Content, Supplementary Material F, http://links.lww.com/PSYMED/B117, we report exploratory correlations with other variables we collected but did not report in this paper.

## DISCUSSION

The present study demonstrates that an individual’s pre-existing somatic symptom burden, and perhaps also their pre-existing symptom priors, greatly influence how easily symptoms can be evoked. Physical activity seems to prevent the activation of symptom priors (“net symptoms”) in the ASP of people with higher pre-existing symptom burdens. We were not able to find any differences between the attention and distraction conditions, except for an unexpected difference in respiratory rates.

As expected, cardiorespiratory activation through cycling produced more event-based, absolute symptoms than resting. Exercise activates the sympathetic nervous system, which causes an excretion of adrenaline and leads to peripheral physiological changes.^65^ These changes last for a while after ceasing activity, which explains why we found heightened symptom reports and arousal directly post-exercise. From a predictive processing perspective, this means that there is more internal noise, explained away by the most available prior (here, the symptom prior), which then leads to increased symptom reports (and particularly so for those with a high level of somatic symptom burden). An interesting avenue for future research would be to administer the ASP *during* an exercise task (eg, by using virtual reality applications) to better study the interplay of induced negative affect and physical activity.

Interestingly, the effects and the “power” of physical activity only become clear when taking pre-existing symptom burden (as measured by the PHQ-15) into account. People with a clinically relevant PHQ-15 score above 10 reported more net symptoms after resting than people below this PHQ-15 score did, but these group differences disappeared in the trials after physical activity. This suggests that physical activity has a beneficial, specific effect on people with higher symptom scores and can sustainably lower their experimentally induced symptoms. Even if the physical activity came first, the effects of physical activity were still visible many blocks later. From a predictive processing perspective, this implies that increased input from physical activity can lower the relative influence of the symptom prior (which is strong in people with higher pre-existing symptom burden) on the posterior-forming process.

We were not able to find support for our hypothesis on attention—people in the attention condition did not have more symptoms than in the distraction condition. Explaining this finding is difficult and points to the complex role of attention and distraction in somatic symptom reporting. Two potential explanations refer to construct validity: firstly, maybe the task only has short-term effects and does not lastingly change symptom experience. Alternatively, perhaps the task was too easy to produce any effects. The mostly high interoceptive accuracy scores (breath-counting accuracy) would support this concern. Going deeper into issues of internal validity, we wonder whether there was perhaps an issue with the nature of tasks: capturing attention to execute a task may also reduce the automatic priming effect of symptom queries, meaning that by measuring attention, we deleted its effects. This is in line with findings from Kósa et al,^33^ who also did not find effects of body-focused attention in a physical performance task. Our inconclusive findings reflect the state of the field of attention towards bodily signals: on the one hand, some studies find that body-focused interventions help people with somatic symptoms,^66,67^ while others do not.^68^ On the other hand, next to manipulating state attention, the trait-level characteristic of interoceptive attention—the degree to which people pay attention to their bodily signals—is associated with heightened somatic symptoms.^26,27^ One could conclude that distraction away from the body would be better for symptom experience, but our findings show no differences between the conditions and do not imply that distraction leads to lower symptom perception, either.

The only difference between attention and distraction conditions was the respiration rate—people in the attention condition breathed less frequently than people in the distraction condition. Slower breathing is also associated with psychophysiological benefits, such as stress reduction, higher heart rate variability, and reduced self-reports of threat-related anxiety.^69–71^ This suggests that while people in the attention condition did not improve on symptom scores, they beneficially adapted their breathing rate, even if they were not explicitly aware of this.

We additionally found that net symptoms decreased over time. To our knowledge, this is the first study that has tested ASP effects over the course of about an hour, as usually studies use the ASP as one task that only lasts ∼20 minutes. For example, in an earlier study, we found no increases or decreases in net symptoms over the course of 9 blocks,^22^ but the current experiment was approximately thrice the length and also showed all stimuli 3 times, which may have led to boredom effects. It would be interesting to investigate whether (occasional) novel stimuli or previously unseen symptom questions also lead to this habituation effect, as oddball events lead to heightened attention and alertness, thus likely engaging participants more.^72^

However, here, too, pre-existing symptom burden had an interesting effect, as it positively predicted symptom reporting per timepoint. This means that people with higher pre-existing symptom burden show less net symptom decrease over time. In addition, the PHQ-15 scores explained over 40% of the variation in intercepts, and thus extend other literature on the ASP that finds that high symptom reporters have higher effects than low symptom reporters.^3,13^

Lastly, end-tidal CO_2_ had a significant negative correlation with the pre-existing symptom burden. This fits the literature showing that people with persistent somatic symptoms often have lower partial CO_2_ pressure at expiration.^73^

From a predictive processing perspective, these findings have multiple implications. We thought that people in the attention condition would be able to “explain away” their symptoms, especially during physical activity phases, because by paying attention to their bodies, they could sample more somatic input or modify their expectations of nonthreatening symptoms explainable by exercise. Meanwhile, we expected people in the distraction condition to be unable to modify their prior and therefore experience the symptoms as more threatening. Interestingly, we saw that people who had a higher pre-existing symptom burden—meaning they had a higher symptom prior—were able to sustainably lower their symptom experiences through physical activity. This means that it was not the somatic input driving the change in symptom experience, as people across the symptom burden spectrum did the same activity. Instead, physical activity seems to have reduced the precision of the prior in people with very precise priors.

From a clinical point of view, this study provides preliminary evidence that encouraging patients with PSS to engage in physical activity might improve their symptom burden. This is in line with a cognitive-behavioral approach to treatment: Changing symptom experience likely needs both cognitive-affective efforts (such as challenging one’s preconceptions about bodily experiences) and behavioral interventions to sample new information about one’s body and symptoms.^74^ Perhaps these interventions do not necessarily have to be exercise, but might also consist of exercises to experience one’s body in new contexts.

To our knowledge, this is one of the first studies that attempted to modify the ASP effect and thereby gain a better understanding of the mutability of somatic symptoms, and the first to do this with physical activation. We also consider our multimethod design, implementing self-reports, behavioral tasks, and physiological measurements, to be a strength. As with any study, there are also some limitations. First, we do not know the exact number of breaths people took in the different conditions, as we worked with average readings every 60 seconds. However, it seems unlikely that people would slow down or change their breathing exactly for the 60-second mark and then speed up again, without the experimenters, who were monitoring the capnograph non-stop, noticing. Secondly, maybe prescribing a certain activity level or a more structured instruction for the activity part would have led to different results—although dose-response relationships in affective sciences in sports are considered outdated^75^ and our manipulation checks were successful, asking participants to do “for them adequate levels” of exercise and allowing them some level of self-selection of intensity may have led to different effects.^76^

Our sample had very few exclusion criteria, and we sampled from a variety of different sources. As a result, it is a mostly young, educated group, but with many different pre-existing conditions. We chose this sampling strategy to increase heterogeneity and external validity compared with a purely student sample, while also allowing for a full spectrum of symptom burden. While we recommend this approach overall, we acknowledge that one drawback is not having exact diagnostic or other medical information.

## CONCLUSION

In conclusion, physical activity seems to buffer against newly induced symptoms in people with higher pre-existing symptom burden by reducing the precision of their priors. This effect lasted at least until the end of the experiment. The role of attention for symptom perception remains elusive, and the question remains how, and which different modalities of attention need to be addressed to improve the posterior symptom experience. The Affect and Symptom Paradigm remains a strong tool to experimentally provoke symptom experience in the lab. To researchers who wish to use the ASP with many repetitions, we recommend implementing occasional novel elements to prevent boredom effects.

## Supplementary Material

## References

[1] Godinho F Frot M Perchet C Magnin M Garcia-Larrea L. Pain influences hedonic assessment of visual inputs. Eur J Neurosci. 2008;27:2219–2228. 18430033 10.1111/j.1460-9568.2008.06196.x

[2] Gerdes ABM Wieser MJ Alpers GW Strack F Pauli P. Why do you smile at me while I’m in pain?—Pain selectively modulates voluntary facial muscle responses to happy faces. Int J Psychophysiol. 2012;85:161–167. 22705169 10.1016/j.ijpsycho.2012.06.002

[3] Bogaerts K Janssens T De Peuter S Van Diest I Van den Bergh O. Negative affective pictures can elicit physical symptoms in high habitual symptom reporters. Psychol Health. 2010;25:685–698. 20204961 10.1080/08870440902814639

[4] Friston K. The free-energy principle: a unified brain theory? Nat Rev Neurosci. 2010;11:127–138. 20068583 10.1038/nrn2787

[5] Pezzulo G Rigoli F Friston K. Active inference, homeostatic regulation and adaptive behavioural control. Prog Neurobiol. 2015;134:17–35. 26365173 10.1016/j.pneurobio.2015.09.001 PMC4779150

[6] Rao RPN Ballard DH. Predictive coding in the visual cortex: a functional interpretation of some extra-classical receptive-field effects. Nat Neurosci. 1999;2:79–87. 10195184 10.1038/4580

[7] Van den Bergh O Witthöft M Petersen S Brown RJ. Symptoms and the body: Taking the inferential leap. Neurosci Biobehav Rev. 2017;74:185–203. 28108416 10.1016/j.neubiorev.2017.01.015

[8] Edwards MJ Adams RA Brown H Parees I Friston KJ. A Bayesian account of ‘hysteria'. Brain. 2012;135(pt 11):3495–3512. 22641838 10.1093/brain/aws129 PMC3501967

[9] Löwe B Toussaint A Rosmalen JGM Huang W-L Burton C Weigel A. Persistent physical symptoms: definition, genesis, and management. Lancet. 2024;403:2649–2662. 38879263 10.1016/S0140-6736(24)00623-8

[10] Witthöft M Bräscher A-K Jungmann SM Köteles F. Somatic symptom perception and interoception. Z Psychol. 2020;228:100–109.

[11] Van den Bergh O Brosschot J Critchley H Thayer JF Ottaviani C. Better safe than sorry: a common signature of general vulnerability for psychopathology. Perspect Psychol Sci. 2021;16:225–246. 33006909 10.1177/1745691620950690

[12] Bogaerts K Millen A Li W De Peuter S Van Diest I Vlemincx E. High symptom reporters are less interoceptively accurate in a symptom-related context. J Psychosom Res. 2008;65:417–424. 18940371 10.1016/j.jpsychores.2008.03.019

[13] Constantinou E Bogaerts K Van Diest I Van den Bergh O. Inducing symptoms in high symptom reporters via emotional pictures: the interactive effects of valence and arousal. J Psychosom Res. 2013;74:191–196. 23438708 10.1016/j.jpsychores.2012.12.015

[14] Van Den Houte M Bogaerts K Van Diest I De Bie J Persoons P Van Oudenhove L. Inducing somatic symptoms in functional syndrome patients: effects of manipulating state negative affect. Psychosom Med. 2017;79:1000–1007. 28914723 10.1097/PSY.0000000000000527

[15] Bogaerts K Rayen L Lavrysen A Van Diest I Janssens T Schruers K. Unraveling the relationship between trait negative affectivity and habitual symptom reporting. PLoS One. 2015;10:e0115748. 25603317 10.1371/journal.pone.0115748 PMC4300148

[16] Petzke TM Weber K Van den Bergh O Witthöft M. Illustrating the Pathway from affect to somatic symptom: the affective picture paradigm. Cogn Emot. 2024;38:801–817. 38411187 10.1080/02699931.2024.2319273

[17] Witthöft M Fischer S Jasper F Rist F Nater UM. Clarifying the latent structure and correlates of somatic symptom distress: a bifactor model approach. Psychol Assess. 2016;28:109–115. 26029944 10.1037/pas0000150

[18] Lamm TT Von Schrottenberg Vv Rauch A Bach B Pedersen HF Rask MT. Five-factor personality traits and functional somatic disorder: a systematic review and meta-analysis. Clin Psychol Rev. 2025;115:102529. 39701015 10.1016/j.cpr.2024.102529

[19] Wilson KE Dishman RK. Personality and physical activity: a systematic review and meta-analysis. Pers Individ Dif. 2015;72:230–242.

[20] Rhodes RE Smith NEI. Personality correlates of physical activity: a review and meta-analysis. Br J Sports Med. 2006;40:958–965. 17124108 10.1136/bjsm.2006.028860 PMC2577457

[21] Sutin AR Stephan Y Luchetti M Artese A Oshio A Terracciano A. The five-factor model of personality and physical inactivity: a meta-analysis of 16 samples. J Res Pers. 2016;63:22–28. 29056783 10.1016/j.jrp.2016.05.001 PMC5650243

[22] Petzke TM Elspaß L Köteles F Van den Bergh O Witthöft M. “And how did that make you feel?”—Repeated symptom queries enhance symptom reports elicited by negative affect. J Psychosom Res. 2024;181:111634. 38453590 10.1016/j.jpsychores.2024.111634

[23] Mehling WE Gopisetty V Daubenmier J Price CJ Hecht FM Stewart A. Body awareness: construct and self-report measures. PLoS One. 2009;4:e5614. 19440300 10.1371/journal.pone.0005614 PMC2680990

[24] Tihanyi BT Sági A Csala B Tolnai N Köteles F. Body awareness, mindfulness and affect: does the kind of physical activity make a difference? EJMH. 2016;11(01-02):97–111.

[25] Barsky AJ. Patients who amplify bodily sensations. Ann Intern Med. 1979;91:63–70. 380428 10.7326/0003-4819-91-1-63

[26] Tünte MR Petzke TM Brand S Murphy J Witthöft M Hoehl S, . He who seeks finds (bodily signals): German Validation of the Interoceptive Attention Scale (IATS) and its Relationship with Subclinical Psychopathology. Journal of Personality Assessment. 2024;106:787–797. 38478969 10.1080/00223891.2024.2316236 PMC7616536

[27] Brand S Petzke TM Witthöft M. The differential relationship between self-reported interoceptive accuracy and attention with psychopathology. Z Klin Psychol Psychother. 2022;51(3-4):165–175.

[28] Treves IN Tello LY Davidson RJ Goldberg SB. The relationship between mindfulness and objective measures of body awareness: a meta-analysis. Sci Rep. 2019;9:17386. 31758073 10.1038/s41598-019-53978-6 PMC6874545

[29] Bornemann B Kovacs P Singer T. Voluntary upregulation of heart rate variability through biofeedback is improved by mental contemplative training. Sci Rep. 2019;9:7860. 31133673 10.1038/s41598-019-44201-7 PMC6536553

[30] Schaefer M Egloff B Gerlach AL Witthöft M. Improving heartbeat perception in patients with medically unexplained symptoms reduces symptom distress. Biol Psychol. 2014;101:69–76. 25038304 10.1016/j.biopsycho.2014.05.012

[31] van de Veer E van Herpen E van Trijp HCM. Body and mind: mindfulness helps consumers to compensate for prior food intake by enhancing the responsiveness to physiological cues. J Consum Res. 2016;42:783–803.

[32] Pennebaker JW Lightner JM. Competition of internal and external information in an exercise setting. J Pers Soc Psychol. 1980;39:165–174. 7411392 10.1037//0022-3514.39.1.165

[33] Kósa L Mikó A Ferentzi E Szabolcs Z Bogdány T Ihász F. Body focus and cardioceptive accuracy are not associated with physical performance and perceived fatigue in a sample of individuals with regular physical activity. Psychophysiology. 2021;58:e13880. 34089192 10.1111/psyp.13880

[34] Kazak AE. Editorial: journal article reporting standards. Am Psychol. 2018;73:1–2. 29345483 10.1037/amp0000263

[35] Rosmalen JGM Burton C Carson A Cosci F Frostholm L Lehnen N. The European Training Network ETUDE (Encompassing Training in fUnctional Disorders across Europe): a new research and training program of the EURONET-SOMA network recruiting 15 early stage researchers. J Psychosom Res. 2021;141:110345. 33385705 10.1016/j.jpsychores.2020.110345

[36] Faul F Erdfelder E Buchner A Lang A-G. Statistical power analyses using G*Power 3.1: tests for correlation and regression analyses. Behav Res Methods. 2009;41:1149–1160. 19897823 10.3758/BRM.41.4.1149

[37] Petzke TM Köteles F Pohl A Witthöft M. Somatic symptom distress is not related to cardioceptive accuracy. J Psychosom Res. 2024;181:111655. 38609776 10.1016/j.jpsychores.2024.111655

[38] Leiner DJ. SoSci Survey [Computer software]. 2019. https://www.soscisurvey.de.

[39] MillisecondInquisit Lab [Computer software]. 2018. https://www.millisecond.com/download

[40] Kroenke K Spitzer RL Williams JBW. The PHQ-15: validity of a new measure for evaluating the severity of somatic symptoms. Psychosom Med. 2002;64:258–266. 11914441 10.1097/00006842-200203000-00008

[41] Han C Pae C-U Patkar AA Masand PS Woong Kim K Joe S-H. Psychometric Properties of the Patient Health Questionnaire–15 (PHQ–15) for measuring the somatic symptoms of psychiatric outpatients. Psychosomatics. 2009;50:580–585. 19996228 10.1176/appi.psy.50.6.580

[42] Stauder A Witthöft M Köteles F. Validation of the Hungarian PHQ-15. A latent variable approach. Clin Neurosci/Ideggyogyaszati Sz. 2021;74:183–190. 10.18071/isz.74.0183 34106550

[43] Löwe B Spitzer RL Zipfel S Herzog W. Gesundheitsfragebogen für Patienten (PHQ-D): Komplettversion und Kurzform, 2nd ed. New York, NY; 2002.

[44] Wientjes CJ Grossman P. Overreactivity of the psyche or the soma? Interindividual associations between psychosomatic symptoms, anxiety, heart rate, and end-tidal partial carbon dioxide pressure. Psychosom Med. 1994;56:533–540. 7871109 10.1097/00006842-199411000-00009

[45] Bradley MM Lang PJ. Measuring emotion: the Self-Assessment Manikin and the semantic differential. J Behav Ther Exp Psychiatry. 1994;25:49–59. 7962581 10.1016/0005-7916(94)90063-9

[46] Lang PJ Bradley MM Cuthbert BN. International Affective Picture System (IAPS): Affective Ratings of Pictures and Instruction Manual: Technical Report A-8. University of Florida; 2008.

[47] Van den Bergh O Stegen K Van de Woestijne KP. Memory effects on symptom reporting in a respiratory learning paradigm. Health Psychol. 1998;17:241–248. 9619473 10.1037//0278-6133.17.3.241

[48] Natarajan A Su H-W Heneghan C Blunt L O’Connor C Niehaus L. Measurement of respiratory rate using wearable devices and applications to COVID-19 detection. NPJ Digit Med. 2021;4:136. 34526602 10.1038/s41746-021-00493-6 PMC8443549

[49] Wahoo fitness. Kickr Snap: Quick Start Guide. 2017. Accessed July 17, 2024. https://support.wahoofitness.com/hc/en-us/article_attachments/4615817902354

[50] Shimano Inc. SHIMANO TOURNEY TZ Multiple Freewheel 3x7-speed [Internet]. 2024. Accessed July 17, 2024. https://bike.shimano.com/en-EU/product/component/tourney/MF-TZ500-7.html

[51] PROACT. Creative PC-900B Handheld Capnograph & Oximeter: Multi Flow Rate Sidestream Technology. 2022. Accessed April 12, 2024. https://www.proactmedical.co.uk/contentfiles/files/PRO20DOC5-1-update_240522_lo.pdf

[52] Hick C Hick A. Kurzlehrbuch Physiologie, 9th ed. Elsevier; 2020:493; p. ger.

[53] IBM Corp. IBM SPSS Statistics for Windows. IBM Corp; 2020.

[54] R Core Team. R: A Language and Environment for Statistical Computing. R Foundation for Statistical Computing; 2013.

[55] Bates D Mächler M Bolker B Walker S. Fitting linear mixed-effects models using lme4. J Stat Soft. 2015;67:1–48.

[56] Kuznetsova A Brockhoff PB Christensen RHB. lmerTest package: tests in linear mixed effects models. J Stat Soft. 2017;82:1–26.

[57] R Core Team. foreign: Read data stored by ’Minitab’, ’S’, ’SAS’, ’SPSS’, ’Stata’, ’Systat’, ’Weka’, ’dBase’. 2022. Accessed June 13, 2022. https://CRAN.R-project.org/package=foreign

[58] Revelle W. psych: Procedures for psychological, psychometric, and personality research. Evanston, IL; 2022. Accessed October 3, 2022. https://CRAN.R-project.org/package=psych

[59] Rosseel Y. lavaan: an R package for structural equation modeling. J Stat Soft. 2012;48:1–36.

[60] Wickham H. Ggplot2: Elegrant Graphics for Data Analysis. Springer; 2016. 1 ressource en ligne. (Use R!).

[61] Schandry R. Heart beat perception and emotional experience. Psychophysiology. 1981;18:483–488. 7267933 10.1111/j.1469-8986.1981.tb02486.x

[62] Wysocki AC Lawson KM Rhemtulla M. Statistical control requires causal justification. Adv Meth Pract Psychol Sci. 2022;5:1–19.

[63] Kroenke K Spitzer RL Williams JBW Lowe B. An ultra-brief screening scale for anxiety and depression: the PHQ-4. Psychosomatics. 2009;50:613–621. 19996233 10.1176/appi.psy.50.6.613

[64] Bagby RM Parker JDA Taylor GJ. The twenty-item Toronto Alexithymia scale—I. Item selection and cross-validation of the factor structure. J Psychosom Res. 1994;38:23–32. 8126686 10.1016/0022-3999(94)90005-1

[65] Goldstein DS. Sympathetic Nervous System. In: Fink G (ed). Encyclopedia of Stress. Elsevier; 2007:697–703.

[66] Fischer D Messner M Pollatos O. Improvement of interoceptive processes after an 8-week body scan intervention. Front Hum Neurosci. 2017;11:1–12. 28955213 10.3389/fnhum.2017.00452 PMC5601051

[67] Schenk L Fischbach JTM Müller R Vögele C Witthöft M Van Diest I. High blood pressure responders show largest increase in heartbeat perception accuracy after post-learning stress following a cardiac interoceptive learning task. Biol Psychol. 2020;154:107919. 32540277 10.1016/j.biopsycho.2020.107919

[68] Parkin L Morgan R Rosselli A Howard M Sheppard A Evans D. Exploring the relationship between mindfulness and cardiac perception. Mindfulness. 2014;5:298–313.

[69] Harris VA Katkin ES Lick JR Habberfield T. Paced respiration as a technique for the modification of autonomic response to stress. Psychophysiology. 1976;13:386–391. 972961 10.1111/j.1469-8986.1976.tb00850.x

[70] McCaul KD Solomon S Holmes DS. Effects of paced respiration and expectations on physiological and psychological responses to threat. J Pers Soc Psychol. 1979;37:564–571. 448632 10.1037//0022-3514.37.4.564

[71] Song H-S Lehrer PM. The effects of specific respiratory rates on heart rate and heart rate variability. Appl Psychophysiol Biofeedback. 2003;28:13–23. 12737093 10.1023/a:1022312815649

[72] Kim H. Involvement of the dorsal and ventral attention networks in oddball stimulus processing: a meta-analysis. Hum Brain Mapp. 2014;35:2265–2284. 23900833 10.1002/hbm.22326 PMC6868981

[73] Niwa S Fila-Pawłowska K Van den Bergh O Rymaszewska J. Respiratory dysfunction in persistent somatic symptoms: a systematic review of observational studies. J Psychosom Res. 2024;181:111607. 38388305 10.1016/j.jpsychores.2024.111607

[74] Kleinstäuber M Thomas P Witthöft M Hiller W. Cognitive Behavior Therapy for Persistent Somatic Symptoms and Somatic Symptom Disorder, 1st ed. Springer; 2025. (Psychotherapie: Manuale).

[75] Ekkekakis P. Pleasure and displeasure from the body: perspectives from exercise. Cogn Emot. 2003;17:213–239. 29715726 10.1080/02699930302292

[76] Parfitt G Hughes S. The exercise intensity–affect relationship: evidence and implications for exercise behavior. J Exerc Sci Fit. 2009;7:S34–S41.

