# Supplementary material for: Physical Activity Decreases Somatic Symptom Distress in the Affect and Symptom Paradigm
Source: Biopsychosoc Sci Med. 2025 Aug 7;87(8):565–75. doi: 10.1097/PSY.0000000000001425 (PMC12490339; doi:10.1097/PSY.0000000000001425)
Supplement: Supplementary file 1 [file psy-87-565-s001.docx]

Supplement A – Materials Used in the Affect and Symptom Paradigm

Based on previous research (unpublished analyses based on the data from Petzke, Weber, et al., 16), we found that the 12 most provoked CSD items were:

- Shivering
- Suffocating feeling
- Need for air
- Pressure on chest
- Rapid heartrate
- Feeling of heat
- Feeling of head warmth
- Hands tremble
- Chest pain around heart region
- Stiffness in fingers or arms
- Pressure or knot in throat
- Faster/deeper breathing than normal

The 24 neutral IAPS pictures were 1114, 1200, 2683, 2692, 2811, 2120, 1302, 1820, 2800, 6212, 6020, 9001, 1932, 9140, 6370, 6550, 9230, 9470, 9181, 9561, 9600, 9911, 9800, and 9410. The neutral pictures were 1121, 1560, 1600, 1670, 1675, 1850, 1942, 1947, 2025, 2102, 2190, 2191, 2272, 2305, 2396, 2480, 5740, 7002, 7004, 7036, 7041, 7205, 7217, and 7546.

Supplement B – Mean valence, arousal, and symptom levels by timepoint

|  |  | Activity first | | | | |  | Rest first | | | | | |
| --- | --- | --- | --- | --- | --- | --- | --- | --- | --- | --- | --- | --- | --- |
|  |  | Distraction (n=46) | |  | Attention(n=32) | |  | Distraction (n=37) | |  | Attention (n=29) | | |
|  |  | M | SD |  | M | SD |  | M | SD |  | M | SD |  |
| Question-naires | PHQ-15 total | 5.59 | 4.13 |  | 6.13 | 3.63 |  | 6.57 | 3.91 |  | 5.62 | 3.90 |  |
|  | HiTOP total | 71.52 | 15.34 |  | 75.56 | 16.40 |  | 73.35 | 15.91 |  | 76.34 | 17.42 |  |
| At baseline | Valence | 6.54 | 1.80 |  | 7.09 | 1.44 |  | 6.38 | 1.62 |  | 7.10 | 1.08 |  |
|  | Arousal | 3.35 | 1.68 |  | 3.38 | 1.96 |  | 3.19 | 1.90 |  | 2.97 | 1.57 |  |
|  | CSD | 15.17 | 2.58 |  | 15.50 | 3.72 |  | 15.81 | 4.36 |  | 14.86 | 2.28 |  |
| First Affect and Symptom Paradigm | Valence neg 1 | 4.72 | 1.77 |  | 5.50 | 1.76 |  | 4.35 | 1.78 |  | 4.76 | 1.81 |  |
|  | Arousal neg 1 | 3.89 | 1.69 |  | 3.72 | 1.67 |  | 3.95 | 1.88 |  | 3.79 | 1.97 |  |
|  | CSD neg 1 | 15.50 | 3.19 |  | 15.56 | 3.67 |  | 16.70 | 4.33 |  | 15.86 | 4.84 |  |
|  | Valence neutral 1 | 6.54 | 1.54 |  | 6.78 | 1.60 |  | 6.54 | 1.37 |  | 6.45 | 1.24 |  |
|  | Arousal neutral 1 | 2.57 | 1.41 |  | 2.81 | 1.60 |  | 2.65 | 1.57 |  | 2.69 | 1.47 |  |
|  | CSD neutral 1 | 13.74 | 2.25 |  | 13.97 | 2.36 |  | 14.11 | 2.87 |  | 13.79 | 2.27 |  |
|  | Valence neg 2 | 4.52 | 1.99 |  | 4.72 | 2.20 |  | 4.19 | 1.90 |  | 4.76 | 1.64 |  |
|  | Arousal neg 2 | 3.85 | 1.92 |  | 3.84 | 2.27 |  | 3.97 | 2.05 |  | 3.48 | 1.55 |  |
|  | CSD neg 2 | 15.04 | 3.03 |  | 15.91 | 3.95 |  | 16.41 | 3.80 |  | 15.31 | 3.51 |  |
|  | Valence neutral 2 | 6.80 | 1.45 |  | 6.75 | 1.39 |  | 6.43 | 1.41 |  | 6.69 | 1.20 |  |
|  | Arousal neutral 2 | 2.43 | 1.38 |  | 2.41 | 1.29 |  | 2.32 | 1.38 |  | 2.76 | 1.79 |  |
|  | CSD neutral 2 | 13.35 | 1.93 |  | 14.00 | 2.33 |  | 14.00 | 2.11 |  | 13.59 | 2.06 |  |
|  | Valence neg 3 | 4.65 | 1.89 |  | 4.84 | 2.02 |  | 4.11 | 2.00 |  | 4.62 | 1.84 |  |
|  | Arousal neg 3 | 3.37 | 1.76 |  | 3.81 | 2.16 |  | 3.92 | 1.88 |  | 3.76 | 1.88 |  |
|  | CSD neg 3 | 14.89 | 3.15 |  | 15.78 | 3.26 |  | 16.51 | 4.01 |  | 15.90 | 5.58 |  |
|  | Valence neutral 3 | 6.59 | 1.50 |  | 7.03 | 1.47 |  | 6.51 | 1.63 |  | 6.45 | 1.09 |  |
|  | Arousal neutral 3 | 2.17 | 1.14 |  | 2.34 | 1.23 |  | 2.51 | 1.59 |  | 2.55 | 1.55 |  |
|  | CSD neutral 3 | 13.41 | 2.19 |  | 13.75 | 1.93 |  | 14.49 | 3.36 |  | 13.72 | 1.96 |  |
|  | Valence neg 4 | 4.35 | 1.88 |  | 4.78 | 2.17 |  | 4.30 | 1.84 |  | 4.79 | 1.82 |  |
|  | Arousal neg 4 | 3.83 | 2.07 |  | 3.69 | 2.18 |  | 3.95 | 2.17 |  | 3.59 | 2.26 |  |
|  | CSD neg 4 | 15.20 | 3.49 |  | 15.63 | 3.54 |  | 17.38 | 5.24 |  | 16.10 | 5.83 |  |
|  | Valence neutral 4 | 6.52 | 1.36 |  | 6.91 | 1.57 |  | 6.59 | 1.46 |  | 6.17 | 1.23 |  |
|  | Arousal neutral 4 | 2.04 | 1.09 |  | 2.28 | 1.46 |  | 2.32 | 1.31 |  | 2.38 | 1.27 |  |
|  | CSD neutral 4 | 13.28 | 1.95 |  | 14.25 | 2.64 |  | 14.16 | 2.90 |  | 13.55 | 2.38 |  |
| Second Affect and Symptom Paradigm | Valence neg 5 | 5.20 | 1.81 |  | 5.56 | 2.00 |  | 5.41 | 1.66 |  | 5.38 | 1.66 |  |
|  | Arousal neg 5 | 3.28 | 1.86 |  | 3.63 | 2.08 |  | 3.08 | 1.53 |  | 2.83 | 1.71 |  |
|  | CSD neg 5 | 15.83 | 3.50 |  | 17.28 | 4.17 |  | 15.35 | 3.45 |  | 14.76 | 3.75 |  |
|  | Valence neutral 5 | 6.70 | 1.44 |  | 6.84 | 1.44 |  | 6.38 | 1.28 |  | 6.41 | 1.24 |  |
|  | Arousal neutral 5 | 2.26 | 1.31 |  | 2.78 | 1.54 |  | 2.41 | 1.38 |  | 2.10 | 1.14 |  |
|  | CSD neutral 5 | 13.96 | 3.02 |  | 15.72 | 3.29 |  | 13.92 | 2.66 |  | 13.62 | 2.08 |  |
|  | Valence neg 6 | 5.15 | 1.80 |  | 5.19 | 2.07 |  | 4.46 | 1.83 |  | 5.10 | 1.72 |  |
|  | Arousal neg 6 | 3.35 | 1.98 |  | 3.66 | 2.10 |  | 3.86 | 2.08 |  | 2.86 | 1.85 |  |
|  | CSD neg 6 | 15.02 | 3.40 |  | 16.34 | 3.94 |  | 16.65 | 5.90 |  | 14.76 | 4.60 |  |
|  | Valence neutral 6 | 6.67 | 1.48 |  | 6.66 | 1.70 |  | 6.24 | 1.48 |  | 6.45 | 1.21 |  |
|  | Arousal neutral 6 | 2.13 | 1.28 |  | 2.75 | 1.72 |  | 2.51 | 1.63 |  | 2.14 | 1.27 |  |
|  | CSD neutral 6 | 13.50 | 2.64 |  | 14.88 | 2.88 |  | 14.49 | 4.20 |  | 13.48 | 2.57 |  |
|  | Valence neg 7 | 4.85 | 1.78 |  | 5.06 | 2.33 |  | 4.68 | 1.83 |  | 5.03 | 1.61 |  |
|  | Arousal neg 7 | 3.41 | 2.04 |  | 3.69 | 2.24 |  | 3.70 | 2.03 |  | 2.83 | 1.51 |  |
|  | CSD neg 7 | 14.35 | 3.04 |  | 15.94 | 3.39 |  | 16.76 | 6.56 |  | 14.55 | 3.22 |  |
|  | Valence neutral 7 | 6.70 | 1.40 |  | 6.88 | 1.29 |  | 6.27 | 1.56 |  | 6.52 | 1.24 |  |
|  | Arousal neutral 7 | 2.26 | 1.51 |  | 2.41 | 1.36 |  | 2.59 | 1.59 |  | 1.97 | 1.12 |  |
|  | CSD neutral 7 | 13.28 | 2.27 |  | 14.09 | 2.64 |  | 14.27 | 3.93 |  | 13.03 | 1.50 |  |
|  | Valence neg 8 | 4.89 | 1.72 |  | 5.31 | 2.10 |  | 4.51 | 1.99 |  | 4.93 | 1.67 |  |
|  | Arousal neg 8 | 3.46 | 1.87 |  | 3.50 | 2.11 |  | 3.68 | 1.99 |  | 2.90 | 1.54 |  |
|  | CSD neg 8 | 14.41 | 2.79 |  | 15.16 | 3.31 |  | 16.24 | 5.90 |  | 15.03 | 4.59 |  |
|  | Valence neutral 8 | 6.76 | 1.45 |  | 7.03 | 1.28 |  | 6.41 | 1.48 |  | 6.34 | 1.32 |  |
|  | Arousal neutral 8 | 2.04 | 1.30 |  | 2.22 | 1.16 |  | 2.68 | 1.65 |  | 2.21 | 1.05 |  |
|  | CSD neutral 8 | 12.93 | 1.73 |  | 13.75 | 2.18 |  | 13.73 | 2.14 |  | 13.24 | 1.64 |  |
| Third Affect and Symptom Paradigm | Valence neg 9 | 5.54 | 1.88 |  | 5.69 | 2.04 |  | 5.22 | 1.89 |  | 5.66 | 1.65 |  |
|  | Arousal neg 9 | 2.93 | 1.68 |  | 3.34 | 2.25 |  | 3.35 | 1.60 |  | 3.00 | 1.44 |  |
|  | CSD neg 9 | 13.96 | 2.16 |  | 14.44 | 2.77 |  | 15.89 | 3.43 |  | 15.10 | 2.86 |  |
|  | Valence neutral 9 | 6.72 | 1.41 |  | 7.06 | 1.44 |  | 6.62 | 1.38 |  | 6.48 | 1.30 |  |
|  | Arousal neutral 9 | 2.13 | 1.45 |  | 2.00 | 1.16 |  | 2.35 | 1.42 |  | 2.31 | 1.11 |  |
|  | CSD neutral 9 | 13.07 | 1.65 |  | 13.66 | 2.18 |  | 13.76 | 2.45 |  | 13.83 | 2.25 |  |
|  | Valence neg 10 | 5.43 | 1.78 |  | 5.59 | 2.08 |  | 5.19 | 1.81 |  | 5.31 | 1.77 |  |
|  | Arousal neg 10 | 3.02 | 1.94 |  | 3.16 | 2.02 |  | 3.11 | 1.70 |  | 2.83 | 1.47 |  |
|  | CSD neg 10 | 13.91 | 2.34 |  | 14.41 | 2.87 |  | 14.95 | 3.50 |  | 14.90 | 4.00 |  |
|  | Valence neutral 10 | 6.48 | 1.41 |  | 7.03 | 1.31 |  | 6.65 | 1.34 |  | 6.52 | 1.30 |  |
|  | Arousal neutral 10 | 2.15 | 1.28 |  | 2.09 | 1.25 |  | 2.16 | 1.12 |  | 2.10 | 1.08 |  |
|  | CSD neutral 10 | 13.17 | 1.84 |  | 13.56 | 2.20 |  | 13.30 | 2.45 |  | 13.52 | 2.38 |  |
|  | Valence neg 11 | 4.98 | 1.84 |  | 5.53 | 2.20 |  | 4.95 | 1.88 |  | 5.14 | 1.55 |  |
|  | Arousal neg 11 | 3.09 | 1.84 |  | 3.34 | 2.18 |  | 3.38 | 1.89 |  | 2.83 | 1.63 |  |
|  | CSD neg 11 | 14.17 | 2.44 |  | 14.22 | 2.37 |  | 15.24 | 3.73 |  | 14.55 | 3.18 |  |
|  | Valence neutral 11 | 6.39 | 1.56 |  | 7.03 | 1.40 |  | 6.41 | 1.52 |  | 6.28 | 1.36 |  |
|  | Arousal neutral 11 | 2.28 | 1.54 |  | 2.13 | 1.13 |  | 2.11 | 1.29 |  | 2.07 | 1.07 |  |
|  | CSD neutral 11 | 13.04 | 1.55 |  | 13.25 | 1.76 |  | 13.68 | 2.71 |  | 13.31 | 1.98 |  |
|  | Valence neg 12 | 5.13 | 1.72 |  | 5.50 | 2.05 |  | 4.92 | 1.91 |  | 5.17 | 1.61 |  |
|  | Arousal neg 12 | 3.35 | 2.11 |  | 3.16 | 2.13 |  | 3.19 | 2.11 |  | 2.76 | 1.55 |  |
|  | CSD neg 12 | 13.98 | 2.36 |  | 14.38 | 2.94 |  | 15.46 | 5.94 |  | 14.55 | 3.27 |  |
|  | Valence neutral 12 | 6.67 | 1.52 |  | 7.19 | 1.28 |  | 6.19 | 1.61 |  | 6.17 | 1.47 |  |
|  | Arousal neutral 12 | 1.80 | 1.07 |  | 2.16 | 1.22 |  | 2.32 | 1.58 |  | 2.24 | 1.27 |  |
|  | CSD neutral 12 | 12.96 | 1.60 |  | 13.47 | 1.76 |  | 14.00 | 4.99 |  | 13.31 | 2.52 |  |
| After activity | Valence | 6.63 | 1.64 |  | 7.09 | 1.42 |  | 6.86 | 1.61 |  | 6.86 | 1.48 |  |
|  | Arousal | 3.54 | 2.00 |  | 3.53 | 1.98 |  | 2.97 | 1.83 |  | 3.07 | 1.71 |  |
|  | CSD | 17.13 | 3.72 |  | 18.44 | 4.24 |  | 16.38 | 3.04 |  | 16.00 | 3.45 |  |
|  | Count | 21.30 | 8.40 |  | 64.28 | 27.14 |  | 20.38 | 6.27 |  | 79.28 | 69.10 |  |
| After rest | Valence | 6.87 | 1.59 |  | 6.88 | 1.88 |  | 6.19 | 1.77 |  | 6.38 | 1.37 |  |
|  | Arousal | 2.22 | 1.43 |  | 2.31 | 1.65 |  | 3.33 | 1.88 |  | 2.34 | 1.42 |  |
|  | CSD | 13.28 | 1.80 |  | 14.16 | 2.89 |  | 15.46 | 3.36 |  | 13.59 | 1.82 |  |
|  | Count | 21.85 | 10.24 |  | 50.59 | 21.99 |  | 20.78 | 5.87 |  | 69.28 | 53.89 |  |

*Note*. CSD = Checklist of Symptoms in daily life; neg = negative (trial); Count: In the distraction conditions, participants were asked to count high notes in a series of tones; in attention conditions, participants were asked to count their breaths.

Supplement C – Additional Analyses As Preregistered

Valence after events did not differ significantly ($F\left( 1.86,262.03 \right)=1.61,p<.20,\eta_{part}^{2}=.01$), and there were no between-person effects of order or attention condition, nor were there interactions between these factors and events (all *p* ≥ .10, all $\eta_{part}^{2}<.02$). For event-based arousal, the timepoints differed in arousal-levels ($F\left( 2,280 \right)=13.68,p<.001,\eta_{part}^{2}=.09$) – people reported least amounts of arousal after resting ($M=2.55,SE=0.14,95\text{\%}CI=$]), but similarly high levels at baseline and after activity ($M_{base}=3.23,SE=0.15,95\text{\%}CI=\left[ 2.93,3.53 \right];M_{act}=3.28,SE=.16,95\text{\%}CI=$]). There were no between-subject effects of order or attention/distraction (all *p* ≥ .52, all $\eta_{part}^{2}\leq.003$). However, there was an event*order interaction, with persons in the activity-first order reporting more arousal after activity and persons in the rest-first condition reporting more arousal after resting.

The physiological measurements were evaluated using rmANOVAs with four timepoints, as these were also measured after the last ASP (Table SC.1). For EtCO_2_, there was a clear timepoint effect ($F\left( 2.27,319.56 \right)=78.03,p<.001,\eta_{part}^{2}=.36$) – participants had the highest EtCO_2_ after cycling, and all pairwise comparisons were significant at *p* < .001 ($M_{act}=39.76,SE=.39,95\text{\%}CI=\left[ 38.99,40.54 \right]$). Order and attention/distraction did not influence EtCO_2_ levels overall (both *p* ≥ .22, both $\eta_{part}^{2}\leq.01$). However, we found a significant event*order interaction ($F\left( 2.27,319.56 \right)=6.84,p<.001,\eta_{part}^{2}=.05$) – people in the activity-first condition had higher EtCO2 at activity than rest-first persons. Additionally, event and attention/distraction interacted significantly ($F\left( 2.27,319.56 \right)=3.45,p=.03,\eta_{part}^{2}=.02$) – however, upon closer inspection, this was solely because of the differences at baseline (see above).

Heartrates were different at each event, with the highest heartrate occurring at physical activity ($F\left( 1.45,228.60 \right)=98.97,p<.001,\eta_{part}^{2}=.41;M_{act}=88.45,SE=1.55,95\text{\%}CI=\left[ 85.38;91.53 \right];$ for comparison $M_{base}=76.46,SE=1.17, 95\text{\%}CI=\left[ 74.15;78.77 \right],M_{rest}=72.49,SE=0.90,95\text{\%}CI=\left[ 70.70;74.25 \right]$). There were no between-person effects, nor were there interactions between events and order or attention/distraction (all *p* ≥ .12, all $\eta_{part}^{2}\leq.016$).

**Table SC.1**

|  | - - - 1. Baseline | - - - 1. While Cycling | - - - 1. While Resting | - - - 1. Endline | Post hoc |
| --- | --- | --- | --- | --- | --- |
| EtCO_2_ | 38.33 (0.30) | 39.76 (0.39) | 37.78 (0.30) | 36.08 (0.31) | 4 < 3^a^ < 1 < 2 |
| HR | 76.46 (1.17) | 88.45 (1.55) | 72.48 (0.90) | 71.35 (0.88) | 4 < 3 < 1^b^ < 2 |
| RR | 14.20 (0.33) | 15.44 (0.35) | 12.52 (0.30) | 12.74 (0.34) | 3 < 4 = 1 < 2^a^ |

*Note*. All post-hoc comparisons were significant at *p* < .001, except those marked ^a^ *p* < .01 and ^b^ *p* < .05. EtCO_2_ =End-tidal CO_2_; RR = respiratory rate; HR = heart rate.

**Supplement D – Randomization Checks**

Participants were compared on all baseline measures (symptoms, valence, arousal, EtCO_2_, BPM, RPM) using t-tests. Regarding order, $n=78$ persons were first instructed to cycle and rested later, while $n=66$ persons had the rest phase first. No significant differences emerged for order (all |*t*| < 1; *df* = 142; all *p* > .36, all Cohen’s *d* < .016).

Regarding the attention vs. distraction between-persons variable, $n=61$ persons were randomized to the attention condition and $n=83$ to the distraction condition. There were no significant differences regarding symptoms, arousal, RPM, and BPM. However, participants differed regarding baseline valence ($M_{dif}=.63,t\left( 142.0 \right)=2.53,p=.013,d=.41$, corrected for heteroscedasticity) and baseline EtCO_2_ ($M_{dif}=1.63,t\left( 142 \right)=2.75,p=.007,d=.46$), with people in the attention condition showing higher values.

Supplement E – Multilevel Analyses

Unconditional Growth Model II (Random Slopes & Intercepts)

**Table SE.1**

*Fixed Effects*

|  | *b* | SE | *p* |
| --- | --- | --- | --- |
| (Intercept) | 0.57 | 0.21 | <.01 |
| Time | -0.09 | 0.02 | <.001 |

Random effects: $\sigma_{0}^{2}=5.29;\sigma_{1}^{2}=0.01;\sigma_{e}^{2}=3.21$

Model fit: AIC = 7354.2, BIC = 7386.9, -2LL=7342.2 df.resid =1722

Conditional Growth Model

**Table SE.2**

*Fixed Effects*

|  | *b* | SE | *p* |
| --- | --- | --- | --- |
| (Intercept) | 0.39 | 0.45 | 0.39 |
| Time | -0.10 | 0.03 | <.01 |
| Attention | -0.06 | 0.58 | 0.92 |
| Order | 0.13 | 0.65 | 0.84 |
| time:attention | 0.01 | 0.04 | 0.85 |
| time:order | 0.01 | 0.05 | 0.75 |
| attention:order | 0.61 | 0.86 | 0.47 |
| time:attention:order | -0.01 | 0.06 | 0.84 |

Random effects: $\sigma_{0}^{2}=5.20;\sigma_{1}^{2}=0.01;\sigma_{e}^{2}=3.21$

**Table SE.3**

*Correlations*

|  | Intercept | time | attention |
| --- | --- | --- | --- |
| Time | -0.693 |  |  |
| Attention | -0.768 | 0.532 |  |
| Order | -0.689 | 0.478 | 0.529 |

Model fit: AIC = 7361.7, BIC = 7427.1, -2LL=7337.7 df.resid =1716

***Model with PHQ-15***

**Table SE.4**

*Fixed Effects*

|  | *b* | SE | *p* |
| --- | --- | --- | --- |
| (Intercept) | -0.29 | 0.43 | .500 |
| time | -0.10 | 0.03 | .004 ** |
| PHQ-15 total score | 0.12 | 0.04 | .005 ** |
| attention | 0.18 | 0.51 | .726 |
| order | -0.22 | 0.56 | .702 |
| Time * attention | 0.01 | 0.04 | .828 |
| Time* order | 0.01 | 0.05 | .781 |
| Attention * order | 0.43 | 0.75 | .563 |
| Time* Attention * Order | -0.01 | 0.06 | .825 |

Random effects: $\sigma_{0}^{2}=3.05;\sigma_{Time}^{2}=0.01;{\sigma_{PHQ-15}^{2}=0.06;\sigma}_{e}^{2}=3.21$

**Table SE.5**

*Correlations*

|  |  |  |  |  |  |  |  | 8. |
| --- | --- | --- | --- | --- | --- | --- | --- | --- |
| 1. Intercept |  |  |  |  |  |  |  |  |
| 1. Time | -.69 |  |  |  |  |  |  |  |
| 1. PHQ-15 | -.43 | .01 |  |  |  |  |  |  |
| 1. Attention | -.73 | .59 | .07 |  |  |  |  |  |
| 1. Order | -.65 | .53 | .05 | .54 |  |  |  |  |
| 1. Time * attention | .53 | -.77 | <.01 | -.77 | -.41 |  |  |  |
| 1. Time* order | .48 | -.69 | <.01 | -.41 | -.77 | .53 |  |  |
| 1. Attention * order | .51 | -.40 | -.09 | -.68 | -.76 | .52 | .58 |  |
| 1. Time* Attention * Order | -.36 | .52 | <.01 | .52 | .59 | -.68 | -.76 | -.77 |

Model fit: AIC = 7321.3, BIC = 7408.6, -2LL=7289.3 df.resid =1712

Supplement F – additional exploratory analyses

**Table SF.1**

*Exploratory correlations with other measures not assessed in the main manuscript*

|  | *M* | *SD* |  |  |  |  |  |  |
| --- | --- | --- | --- | --- | --- | --- | --- | --- |
| 1. PHQ-15 | 5.97 | 3.90 |  |  |  |  |  |  |
| 1. HiTOP-SF | 73.86 | 16.10 | .616^***^  [.50; .71] |  |  |  |  |  |
| 1. SSD-12 | 9.74 | 8.42 | .621^***^  [.51; .71] | .776^***^  [.70; .83] |  |  |  |  |
| 1. HAI | 26.82 | 11.43 | .480^***^  [.34; .60] | .738^***^  [.65; .80] | .791^***^  [.72; .85] |  |  |  |
| 1. BPQ | 33.98 | 11.93 | .051  [-.11; .21] | .186^*^  [.02; .34] | .094  [-.07; .25] | .097  [-.07; .26] |  |  |
| 1. TAS | 42.47 | 11.08 | .318^***^  [.16; .46] | .258^**^  [.10; .40] | .371^***^  [.22; .50] | .399^***^  [.25; .53] | -.026  [-.19; .14] |  |
| 1. PHQ-4 | 3.08 | 2.49 | .554^***^  [.43; .66] | .412^***^  [.27; .54] | .444^***^  [.30; .57] | .400^***^  [.25; .53] | .050  [-.11; .21] | .439^**^  [.30;.56] |

*Note*. PHQ-15: Patient Health Questionnaire 15; HiTOP-SF: HiTOP somatoform candidate items (1) in a German version (2); SSD-12: Somatic Symptom Disorder B-Criteria Scale (3); HAI: Health Anxiety Inventory (4) in its German version (5); BPQ: Body Perception Questionnaire (6) in its shortened version by Cabrera and colleagues (7); TAS: Toronto Alexithymia Scale (8) in its German validation (9); PHQ-4: Patient Health Questionnaire 4 (10) in its German validation (11).

* *p* < .050; ** *p* < .010; *** *p* < .001

References

1. Sellbom M, Forbush KT, Gould SR, Markon KE, Watson D, Witthöft M. HiTOP Assessment of the Somatoform Spectrum and Eating Disorders. Assessment. 2021;10731911211020825. doi:10.1177/10731911211020825 Cited in: PubMed; PMID 34105380.

2. Hartmann J, Bräscher A-K, Forbush KT, Sellbom M, Watson D, Witthöft M. The Somatoform Spectrum Within the Hierarchical Taxonomy of Psychopathology System: A Taxometric Test of the Latent Structure. Psychosom Med. 2022;84(9):1067–76. doi:10.1097/PSY.0000000000001105 Cited in: PubMed; PMID 35797578.

3. Toussaint A, Murray AM, Voigt K, Herzog A, Gierk B, Kroenke K, et al. Development and Validation of the Somatic Symptom Disorder-B Criteria Scale (SSD-12). Psychosom Med. 2016;78(1):5–12. doi:10.1097/psy.0000000000000240 Cited in: PubMed; PMID 26461855.

4. Salkovskis PM, Rimes KA, Warwick HMC, Clark DM. The Health Anxiety Inventory: development and validation of scales for the measurement of health anxiety and hypochondriasis. Psychol Med. 2002;32(5):843–53. doi:10.1017/s0033291702005822 Cited in: PubMed; PMID 12171378.

5. Bailer J, Witthöft M. Deutsches modifiziertes Health Anxiety Inventory (MK-HAI); 2006. deu.

6. Porges SW. Body Perception Questionnaire: University of Maryland; 1993.

7. Cabrera A, Kolacz J, Pailhez G, Bulbena‐Cabre A, Bulbena A, Porges SW. Assessing body awareness and autonomic reactivity: Factor structure and psychometric properties of the Body Perception Questionnaire‐Short Form (BPQ‐SF). Int J Methods Psychiatr Res. 2017;27(2). doi:10.1002/mpr.1596 Cited in: PubMed; PMID 29193423.

8. Bagby RM, Parker JD, Taylor GJ. The twenty-item Toronto Alexithymia scale—I. Item selection and cross-validation of the factor structure. Journal of Psychosomatic Research. 1994;38(1):23–32. doi:10.1016/0022-3999(94)90005-1

9. Bach M, Bach D, Zwaan M de, Serim M, Böhmer F. Validierung der deutschen Version der 20-Item Toronto-Alexithymie-Skala bei Normalpersonen und psychiatrischen Patienten [Validation of the German version of the 20-item Toronto Alexithymia Scale in normal persons and psychiatric patients]. Psychother Psychosom Med Psychol. 1996;46(1):23–8. ger. Cited in: PubMed; PMID 8850096.

10. Kroenke K, Spitzer RL, Williams JBW, Löwe B. An ultra-brief screening scale for anxiety and depression: the PHQ-4. Psychosomatics. 2009;50(6):613–21. doi:10.1176/appi.psy.50.6.613 Cited in: PubMed; PMID 19996233.

11. Löwe B, Wahl I, Rose M, Spitzer C, Glaesmer H, Wingenfeld K, et al. A 4-item measure of depression and anxiety: validation and standardization of the Patient Health Questionnaire-4 (PHQ-4) in the general population. Journal of Affective Disorders. 2010;122(1-2):86–95. doi:10.1016/j.jad.2009.06.019 Cited in: PubMed; PMID 19616305.
